# A University and Community-Based Partnership: After-School Mentoring Activities to Support Positive Mental Health for Children Who Are Refugees

**DOI:** 10.3390/ijerph19106328

**Published:** 2022-05-23

**Authors:** Laura A. Nabors, Tina L. Stanton-Chapman, Filiberto Toledano-Toledano

**Affiliations:** 1 College of Education, Criminal Justice and Human Services, University of Cincinnati, Cincinnati, OH 45221, USA; naborsla@ucmail.uc.edu; 2 Unidad de Investigación en Medicina Basada en Evidencias, Hospital Infantil de México Federico Gómez National Institute of Health, Márquez 162, Cuauhtémoc, Mexico City 06720, Mexico; filiberto.toledano.phd@gmail.com; 3 Unidad de Investigación Sociomédica, Intituto Nacional de Rehabilitación Luis Guillermo Ibarra Ibarra, Calzada México-Xochimilco 289, Arenal de Guadalupe, Tlalpan, Mexico City 14389, Mexico

**Keywords:** mentoring, after-school, mental health, reflection, refugees

## Abstract

The objective of this study was to examine mentors’ perceptions of a pilot service-learning program designed to provide activities to promote the self-esteem and positive development of elementary school-age children who were refugees. Activities were designed to promote self-esteem, self-confidence, social skills development, and problem-solving. College students completed reflection journals to record their perceptions of mentoring and what the children were learning and experiencing. The results indicated that mentors believed the children were learning ideas to improve their self-esteem and social development. Mentors’ impressions were corroborated by reports about the program from staff who worked with the children daily. Involving parents in programming, may have extended the reach of program efforts. Some children may have benefited from evaluations to determine if counseling would benefit them, given the trauma history they and their family members were potentially facing. This was a pilot program implementation study, and a limitation is that data from youth and parents about mental health outcomes were lacking. In the future, assessing perceptions of children, involving their caregivers in programming, and then assessing their caregivers’ perceptions of the impact of the program on children’s self-esteem and social and emotional functioning will provide critical information about program success and information for program development.

## 1. Introduction

### 1.1. Activities to Support Positive Mental Health for Children Who Are Refugees

Children who are refugees or children who reside in families where parents are refugees face many challenges, related to isolation, acculturation, or being displaced, which can negatively affect their social and emotional functioning [1]. Children can face language barriers that make it difficult to learn and connect with peers, and indeed, language barriers can be a “family experience” that in turn, may be associated with poverty and poor access to health care services, including mental health services [2]. Additionally, displaced children can be recovering from trauma or a web of complex trauma, as they have experienced many interwoven traumas in a repeating fashion related to war, famine, violence, and/or discrimination [2]. Exposure to trauma and family stress places children at-risk for mental health problems such as depression and anxiety. Improving positive social support for children can lessen feelings of sadness, possible depression, and enhance school functioning [3]. Frounfelker et al. [4] indicated that the numbers of refugees continue to rise and that addressing mental health concerns is paramount for improving child functioning and development. Bettman et al. [1] recommended that schools devote resources to helping these children. However, there are a dearth of studies examining the impacts of support programs designed to enhance self-esteem and promote mental health of children who are refugees.

### 1.2. Schools as a Hub for Services

Schools can play a central role in facilitating children’s adjustment while also promoting mental health and the psychosocial well-being of children as they adjust to residing in a new land with a new culture. Schools can be a center for teaching the new language and culture. At the same time, schools can be a place for affirming the child’s and family’s home culture and respecting their needs for continuity of culture, as keeping in touch with former cultural practices can be supportive for families [5]. Hence, supportive school-based programs that foster positive emotional functioning could assist with mitigating fears and creating a safe, nurturing environment where children can have positive experiences that remind them that their environment can be a safe and supporting space. Ensuring that young children have opportunities to learn about positive mental health might provide them with new coping strategies to promote their self-concepts and enhance their adjustment at school [4,5]. Engaging in this type of programming could benefit connection and involvement in school, which can be beneficial because children residing in families that have been displaced are at risk for isolation from others and for poor academic outcomes [6].

### 1.3. Promoting Positive Mental Health in School Settings

A guiding idea for this work was that it is important to allow children to participate in activities that address aspects of positive mental health, such as positive self-concept and improving self-esteem, to promote social and emotional functioning [7]. Improving positive self-concept, increasing involvement in positive activities, and providing young children who are refugees with improved self-esteem could serve as a protective factor for positive social and emotional development. Castañeda et al. [8] proposed a framework that focuses on helping individuals improve well-being through fostering resilience and building personal strengths—cornerstones of our children’s group. Frounfelker et al. [4] proposed that mentoring and school-based activities to promote positive mental health can support children who are refugees, and they delivered positive mental health activities in an after-school group using supportive college mentors; the authors reported that culturally sensitive mentoring programs have the potential to strengthen youth development and improve children’s sense of belonging in their schools. To better understand how mentoring and school-based activities promote resilience and positive mental health in children who are refugees, this qualitative case study examined the utility of an after-school mentoring program in supporting the social and emotional development, emotional well-being, and positive mental health of program participants. The first goal was to describe the mentoring project and demonstrate how its activities provided authentic, participatory, and meaningful social-emotional learning for child participants. Although program impact was not evaluated in the current study, those invested (i.e., university partners, community partners) did want to know if the college students felt they could implement the activities and lesson plans and whether the parents, grandparents, and children valued the activities and lesson plans (second goal).

## 2. Method

This implementation project [9,10] examined the types of mentoring and school-based activities and refection evident in an after-school group designed to promote resilience and positive mental health in children who were refugees. A qualitative case study was an appropriate methodology for uncovering and explicating the meaning and action of mentor (teacher) reflection [10]. Reflection is a theoretical practice that is often encouraged in the teacher-coaching literature [11] and is one of the most significant ways teachers examine and change their practice [12]. Typically, teacher reflection is a tool for improving planning for future practice. The current study was a pilot study to evaluate the feasibility of mentoring and after-school activities to boost self-esteem and use of mentor (teacher) reflection as a means to understanding mentors’ perceptions of their practices and to their reviewing their beliefs about the impact of their teaching.

### 2.1. Context for Children

The children for this pilot were children who were refugees from Bhutan or children whose parents were refugees from Bhutan. Their parents had fled Bhutan either due to political and/or ethnic persecution. The children or their parents had spent time in a refugee camp, most often in Nepal. Many of the parents had experienced trauma, related post-traumatic stress, depression, and anxiety. The children had sometimes directly experienced trauma or were at risk for experiencing intergenerational trauma as their parents relived and coped with their trauma-related experiences in their new home. Intergenerational trauma includes their parents’ memories of camps, war, and their reactions to being refugees (i.e., being separated from family, displaced in camps, moving to new countries) [2].

The children and their families were now residing in the United States Midwest. The families from Bhutan are more likely to be low income, lack significant educational experiences, and have low English proficiency.

### 2.2. Setting

A Midwestern after-school program served as a purposefully selected site [13] for examining mentors’ reflections. The university-community partnership created a significant collaboration focused on positive social and emotional development [14]. The children who were refugees from Bhutan or whose parents were refugees from Bhutan were enrolled in the after-school program prior to the collaboration with the university.

A university professor met with the program’s after-school director to develop a program whose purpose was to strengthen the self-esteem of elementary school-age children who were refugees. A critical program goal was to empower school-aged children and their families through tutoring, mentorship, and leadership development. The director selected one after-school program for mentoring purposes. Since status as displaced places a child into the at-risk category, the director of the school district’s after-school program hoped to provide as many preventative mental health and academic services as possible. 

The first author was assigned an elementary school. The director from the after-school program met with the first author to discuss details. After learning about the children in the after-school program, the first author decided to target social-emotional skills and recruit mentors from her undergraduate course in global health.

### 2.3. Participants

In an effort to document and describe the positive mentoring in place at this after-school program, teacher reflection data were collected from mentors. Seven college students participating in service-learning served as mentors in the after-school program. They were led by a graduate student and the first author while at the program site.

The mentors worked with the children in the after-school program weekly for 9 weeks in 60-min sessions. The undergraduate students were enrolled in a global health course with a service-learning requirement. Student participation in the mentorship program and evaluation process was voluntary.

Mentors ranged in age from 19 to 22 years. There were three females and four males. Their race/ethnicity included one African American and six Caucasian students. All students were undergraduates enrolled in a bachelor of science in health promotion and education program. Students enrolled in this program take an array of courses (e.g., global health, mental health issues, adolescent and child health and development, health issues of vulnerable and marginalized populations). The graduate student was enrolled in a master’s program for health education. He was from Turkey. The college professor for the global health course was a child psychologist with over 20 years practicing in the field, who was Caucasian and female.

### 2.4. Procedure

#### 2.4.1. Activity Topics

Prior to the start of the after-school program, the mentors met with the program staff of the after-school program and the college professor who was teaching the global health course with an optional service-learning requirement. The purpose of these meetings was to design activities for the nine weeks of the after-school program. Table 1 presents a list of the activity topics developed in the university-community partnership meetings and addressed across the weekly sessions. The college professor developed and created the activity topics. The topics covered stress management, conflict resolution, improving self-esteem, and improving emotional expression and social skills [15].

#### 2.4.2. Mentor Training

Mentors met with their college professor for weekly supervisor meetings and service-learning instruction. Weekly supervision meetings consisted of developing a detailed lesson plan based on the previously approved activity topics listed in Table 1. Lesson plans were designed as hands-on activities that were conducted to teach various social and emotional skills (promoting positive mental health and self-esteem).

Service-learning training permitted college student mentors opportunities to gain real-world experience teaching young children. Specifically, they were taught strategies on how to teach children social skills and conduct activities that enhance child emotional functioning [16]. Mentors also learned more about refugee lifestyles and gained knowledge of their cultural backgrounds [17,18].

Mentors were taught how to use reflection journals in meetings with their college professor. Reflective teaching “involves a recognition and examination over implications of one’s beliefs and experiences, as well as the opportunities and constraints provided by the social conditions in which the teacher works” [19] (p. 20). Similar to reflective teaching, mentors were asked to reflect on their impression of the impact of the lesson plan and corresponding activities for children on their social and emotional functioning, self-esteem, and cultural appropriateness. They were also asked to reflect on the impacts of the activities on their abilities as leaders and competence in their practice.

#### 2.4.3. Lesson Plan Implementation

Each mentor was assigned one to two children to mentor during the group meetings. Twelve children attended weekly meetings with their assigned mentor. Children ranged in age from 6 through 11 years (first through fifth grades). A total of 16 children participated in the after-school mentor program. Each weekly session typically lasted for one hour and had three components: (a) a rapport building activity; (b) a lesson of the day—a positive mental health activity (e.g., stress management, conflict resolution, improving self-esteem); and (c) a review of the activity.

The mentoring session opened with a rapport-building activity (see Table 1). Rapport-building activities occurred during the first 15 min of the session with the intent of establishing a mutually trusting and respectful relationship between the mentors and children [20]. Examples of activities included playing games, sharing one positive thing about your day, and squiggle-scribble drawings. In the squiggle-scribble drawing activity, the child and mentor took turns drawing a squiggle-scribble line and connected it to their partner’s squibble-scribble line until they decided to guess what type of figure, object, or scene emerged from their scribble lines.

Once the rapport-building activity ended, the lesson-of-the-day activity began (see Table 1). Lesson of the day activities were designed to promote positive mental health and self-esteem in young children and lasted 30 to 35-min in length. The purpose of the lesson-of-the-day activities was to teach social and emotional skills as positive mental health activities (e.g., stress management, conflict resolution, improving self-esteem, improving emotional expression and social skills) [15]. An example of a lesson of the day activity was Anger Eater Bags. For this activity, the child received a paper bag, crayons, glue, yarn, and 12 small note cards. The mentor directed the child to draw six angry faces on six note cards. The mentor then directed the child to draw six happy faces on the six remaining note cards. Next, the mentor directed the child to cut a hole for a mouth in the bag and decorate the bag to make a face on it (around the mouth). The top of the open bag was the head or the “thinking place where one solves problems” The mentor then put a paper clip with an angry face slip of paper in the top of the bag and said, “I think about my anger and how to solve it in a peaceful way.” The mentor then put a slip of paper with a happy face on it through the paper bag mouth and said, “I say what my feelings are and say positive things to others.” The mentor taught the child that when upset rather than saying angry words, one could say positive words. The child and mentor then practiced expressing anger in positive ways (e.g., “I feel upset. I will take three deep breaths to calm down. I will tell someone how I feel and about what is upsetting me”). The mentor then asked the child to practice turning angry face note cards into positive thoughts. When the child had a positive solution, he/she stated the solution positively, and then the child placed the happy note card into the anger eater’s mouth. Mentors served as role models, reviewing calming breaths, counting to 10 and relaxing, and then using words to express one’s upset feelings. There were other lesson plans to promote self-esteem, problem-solve, express emotions, and develop a positive sense of self.

The final component of the lesson plan implementation was the review of the activity. In the review of the activity, the mentors and the children reviewed the activity and lesson plan with the parents and/or grandparents before the family departed the after-school program. This component was 5 to 10 min in length. The purpose of reviewing the activity and the lesson plan was to connect the parents and grandparents with the after-school program through positive communication and to hopefully reinforce the positive mental health concepts taught earlier in the session and establish a parent-school partnership.

### 2.5. Data Analysis

Coders analyzed journals to find meaningful points of learning for children by searching for words such as activities that worked or did not work and mentor impressions of program satisfaction. This step was coined code definition [13]. Coders determined codes or themes based on consensus. Next, the individual words or phrases highlighted from the mentors’ journals were tentatively placed into categories and/or subcategories to illustrate themes. Next, coders examined possible connections among themes and determined if there were subthemes. Finally, the coders connected themes to the literature and theory. Two examples of the analytic induction method conducted on mentor journals are as follows:

*For the Anger Eater Bag Activity, I don’t think many families will have the time to create this type of activity. Maybe the parent and child can hug themselves and take five deep breaths together to calm down. I would recommend this as a follow–up*. (FOLLOW-UP)

*Whenever I ask* [name of child] *about what she did with her friends on the playground during recess, she doesn’t answer. She starts rocking back and forth. She is always talkative until I mention the word playground. Then she gets quiet and starts rocking. Something about the playground makes her anxious. She may need to talk to someone about it.* (ASSISTANCE)

Coders used analytic induction [21], and their first goal was to describe the mentoring project and demonstrate how its activities provided authentic, participatory, and meaningful social-emotional learning for the child participants. While program impact was not evaluated in the current study, those invested (i.e., university partners, community partners) did want to know if the college students felt they could implement the activities and lesson plans and whether the mentors believed that the parents, grandparents, and children valued the activities and lesson plans (second goal). Analytic codes developed across the mentor journals included the following: (a) review of activity—follow-up, adaptation, suggestion, alternative, satisfaction, and positive; (b) children may need more assistance—assistance, screening, and behavior; and (c) mentors creatively modified lesson plans as needed—modify.

### 2.6. Findings

Three key findings were evident across the mentor (teacher) reflections and are explicated in the subheadings below. First, mentor reflection journals discussing the review of the activity component of the after-school program noted satisfaction and suggestions for improvement. Second, the mentors came to an understanding about the children’s needs during this after-school program and realized the children may need more professional assistance than what the after-school program provided. Third, the mentors creatively modified the lesson plans to work with the children when they realized the current plan was not working.

#### 2.6.1. Review of Activity Component

It is evident across the mentor (teacher) reflections that mentors did indeed review the activity and lesson plan with the parent or grandparent when he/she came to pick up his/her child. Mentors shared suggestions for developing follow-up lesson plans for families that would enhance programming efforts. Suggestions included that the activities could be adapted so that the families could do the activities in the home, offered alternative ideas if the activities seemed too elaborate or too complicated to be continued in the home setting, or provided suggestions for follow-up questions caregivers could ask their children about the activity that took place in the mentoring program. The community partners mentioned to the mentors that they were satisfied with the activities and the lesson plans. They also reported that the children enrolled in the after-school program showed more positive attitudes at school by the end of the school year, which they attributed to the mentoring program.

#### 2.6.2. Children May Need More Assistance

The mentors, in their journal reflections, realized that the after-school program may not be sufficient to meet all of the children’s needs. A few journal reflections mentioned children’s specific behaviors (e.g., rocking back and forth), and one child told a mentor he was nervous), which could have indicated the child was experiencing possible anxiety or depression. Several journal reflections recommended a need for mental health screenings in the schools given the “at-risk” status of children who are refugees. Three important areas for screening included depression, anxiety, and symptoms of post-traumatic stress.

#### 2.6.3. Mentors Creatively Modified Lesson Plans as Needed

Mentors creatively modified the activities and lesson plans to work with children. In other words, they worked to understand the children’s needs and planned their future instruction accordingly. For example, telling a positive story was modified to developing a comic “to make others laugh.” The mentors quickly learned students’ interests and preferences and determined ways to modify activities based on these interests and preferences for better student buy-in.

## 3. Discussion

The results of this study corroborate findings from previous research and hold several important implications. Our first finding—of satisfaction with the after-school mentoring and suggestions for developing follow-up lesson plans for families—illustrates the necessity and importance of parent-mentor communication and collaboration. Mentors consistently noted that developing lesson plans for parents and grandparents would strengthen programming efforts. Indeed, prior literature demonstrates that creating lesson plans for parents (e.g., book in the bags, discovery bags) increases parental understanding of their children’s skills, enthusiasm resulting from their children’s growth, and enjoyment of the activities and shared time together [22,23,24]. Research on child progress from parent-implemented lesson plans within naturalistic settings show positive development [22,24,25], especially with skills children were taught in an early childhood classroom. Future iterations of the after-school mentor program should include follow-up lesson plans for parents and grandparents so they can continue to work on previously taught concepts at home.

Second, this study revealed that the after-school program might not be sufficient to meet all of the children’s needs. Several journal reflections recommended a need for mental health screenings in the schools. Three important areas for screening include depression, anxiety, and symptoms of post-traumatic stress. If children display significant symptoms, then a referral to a school psychologist or school counselor may be necessary. This response strategy is similar to that of the Response to Intervention model [26] that is widely used in early childhood special education and Positive Behavior Support in Schools as a decision-making process for problem-solving in evidence-based interventions. Considering this model, all children are screened for mental health disorders (e.g., depression, anxiety). Children who receive clinical ratings on screening measures would receive referrals to a school psychologist or school counselor and most likely be eligible for Tier 2 and/or Tier 3 intervention services. Symptoms, based on the authors’ experience, signaling a possible need for referral are presented in Table 2.

Third, mentors creatively modified the activities and lesson plans to work with the children. Research indicates that it is challenging for beginning teachers to monitor student learning and modify instruction [27]. It is possible that the types of activities and lesson plans in the current study allowed the mentors to use their intuition and insight when content needed to be changed. While this instructional decision-making is not best practice in actual classroom settings [28], it appears to work in an after-school mentoring program. This study also draws attention to reflective teaching, which has been emphasized in recent years [29]. For example, mentors asked if they could take children to the lunchroom, where some of the children were able to get snacks provided to a different group of children by another after-school program at the school. The mentors, in their journals, thought their mentees could interact with other peers, increasing their social networks.

## 4. Limitations

The findings of our study should be viewed with caution given several limitations. First, this was a pilot study. The after-school mentoring program was a single-site operation whose purpose was to evaluate the feasibility of the mentoring and after-school-based activities and the use of mentor (teacher) reflection as a means to change practice. Second, there were no scientific data about the program’s impact on the children’s mental health functioning or school performance. Previous research has shown that similar activities and lesson plans had positive impacts for children who were homeless [30,31]. Anecdotal reports from community partners indicated that programming and collaborating with mentors were positive experiences for the child participants. Third, mentors and community partners did not provide parent training. Adding services for parents, through home-visiting or outreach through the internet, can be an avenue for parents to learn skills for promoting their children’s social and emotional development.

### Future Work and Implications for Researchers and Policymakers

Future work should focus on developing interventions that are broader in scope and capture specific issues for families, including oppression of families and understanding family narratives. These narratives can involve both children and their parents and include supportive sessions to enhance family relationships and to improve the mental health of parents and children. Searching for strengths of children and families and connecting with families is a positive way to build rapport and reduce feelings of being a burden that some parents may harbor. Enhancing cross-cultural competence, to understand the backdrops of families’ lives and understanding their social contexts, may be another important tool for meeting the children and families “where they are” and connecting with them. Working to understand cultural differences and share positive ideas can further child and parent integration into their communities and help them develop a sense of “belonging in” and “connecting to” their new communities. Health professionals also need to be aware of special needs and the impact of trauma. Referral sources and resources need to be in place for those who may be experiencing anxiety or sadness or reexperiencing trauma that occurred in previous situations. Health professionals need to think broadly and work with local organizations and hospitals to care for health, nutrition, educational, financial, and mental health needs of children and families. They also need to think about community, systems, and policy-level changes that will help families on broader levels [32].

Linton, Green, and the Council on Community Pediatrics [33] reported that improving community support for children residing in families who are immigrants is important for engaging and supporting families. This group also recommended finding system-level support for children and families, and the school can be one of these systems. Children and parents will have contact with schools, and schools are a center for education, and if the school is a full-service school (either containing or linking to other services), then health care and mental health care services can be accessed. After-school programs, and perhaps programming during the school day, can be used to promote social and emotional development of children. Inviting “near peers” such as high school or college students to work with young children provides positive experiences for the mentor and the mentee. It can be a cost-effective way to have a “work force” to deliver social and emotional curricula to young children. Providing interventions with multiple components, such as academic tutoring for child and family outreach services (e.g., case managers and mental health professionals) to address child and family needs will be important [4]. It is critical to view children and families as resilient and as moving forward in their new lives. Supporting their acculturation as well as cultural practices from their home countries could provide bridges to the future and support from the past to help children and families become happy and productive in their new lives.

## 5. Conclusions

The objective of this study was to examine mentors’ perceptions of a pilot service-learning program designed to provide activities to promote the self-esteem and positive development (e.g., mental health) of elementary school-age children who were refugees. Activities were designed to promote self-esteem, self-confidence, social skills development, and problem-solving. College students completed reflection journals to record their perceptions of mentoring and what the children were learning and experiencing. Results indicated that the mentors believed the children were learning ideas to improve their self-esteem and social development. Three key findings were evident across the mentor (teacher) reflections: (a) mentor reflection journals noted satisfaction and suggestions for improvement; (b) the mentors realized the children may need more professional assistance than what the after-school program provided; and (c) the mentors creatively modified the lesson plans to work with the children when they realized the current plan was not working.

## Figures and Tables

**Table 1:** Activities for Children to Address Positive Mental Health and Self-Esteem.

Area	Activities in the Area
Establishing Rapport with Children	Joint Storytelling (to build sharing and positive regard) Feelings worksheets Squiggle—Scribble Drawings My day was… how was your day? (sentence completion activity) Games
Positive Mental Health	Feelings Charades (children make faces to express feelings and children try to guess the feelings; promotes recognition and expression of emotion) Word searches to find different feelings words (discuss the meaning of the words and provide examples for children) Troubles Bubbles (draw a trouble bubble and draw the troublesome situation or worry in the bubble and then discuss the issue; one-on-one activity to share troubles or bothersome feelings) Paper Bag Trash Eaters (Anger Management Activity) Role Plays (to solve different social dilemmas with the advice machine)
Self-Esteem	Nice Things about Me Books Becoming an Expert (discussing areas of expertise for children or talents) Shield of Strength (draw a shield and draw favorite activities and write positive things about the child on the shield) Nice Things about Me Bags (decorate a paper bag and add slips of paper inside with nice things about the child and his or her talents) “Helping Others Jar” with cards for things to do for others Self-Affirmation Calendars (write one positive thing about the child each day)

**Table 2:** Symptoms Signaling Possible Need for Referral for Counseling.

Type of Mental Health Problem	Symptoms
Depression	sadness, change in eating pattern (over or undereating), weight loss or gain, change in sleeping habits (too much or too little sleep), worry, withdrawal from others and lack of involvement in daily activities, feelings of hopeless (lack of hope for the future)
Anxiety	repetitive worry, fears, hypervigilance (startle easily and/or often), perfectionist tendencies, feeling “I’m never good enough, expectations of fear that are significant given the situation
Post-Traumatic Stress Symptoms	repeating, intrusive worries over previous traumatic events (e.g., “flashbacks”), repetitive bad dreams, high anxiety, hypervigilance, avoidance of feared situations or situations similar to traumatic events, mood changes

*Note*. Symptoms reach referral level if they interfere with daily activities and/or there are several symptoms that persist over time. Additionally, symptoms in different categories can overlap, and the list of symptoms in Table 2 is not exhaustive (there may be other symptoms identifying the mental health problems presented in this table).

## Data Availability

Data coding of mentor interviews is available from Laura Nabors; email: naborsla@ucmail.uc.edu.
